# Development of chimeric HIV Env immunogens for mucosal delivery with attenuated canine distemper virus (CDV) vaccine vectors

**DOI:** 10.1186/1742-4690-9-S2-P298

**Published:** 2012-09-13

**Authors:** X Zhang, S Richlak, HT Nguyen, O Wallace, G Morrow, M Caulfield, C Parks

**Affiliations:** 1 International AIDS Vaccine Initiative, Brooklyn, NY, USA

## Background

Our aim is to develop replication-competent viral vectors for mucosal delivery, which express HIV-Env immunogens that closely mimic the trimeric glycoprotein spike found on HIV particles. We have developed attenuated recombinant CDV (rCDV) expressing SIVmac239-Env and shown that this vector can be used safely to elicit Env-specific immune responses in ferrets and non-human primates through intranasal administration.

## Methods

To augment the cell surface expression of trimeric HIV-Env and increase the replicative capacity of rCDV vectors encoding the HIV glycoprotein, we have constructed chimeric immunogens in which signal peptide (SP), transmembrane domain (TM), or cytoplasmic tail (CT) domains in HIV-Env have been replaced with analogous sequences from the vesicular stomatitis virus (VSV)-G or CDV-F glycoproteins and compared cell surface protein expression, antibody binding profiles and Env function in transient expression assays.

## Results

Chimeric glycoprotein based on subtype C Env proteins were expressed on the surface of transfected cells in conformations recognized by various broadly neutralizing antibodies (bnAb) targeting distinct Env regions including VRC01, b12, and b6 specific for the CD4 binding site, PG9 and PG16 (V1/V2), 4e10 (gp41) but not by PGT-126 (V3) and 2G12 (glycans). Moreover, treatment of transfected cells with soluble CD4 induced conformational changes needed to expose epitopes for CD4 binding-dependent antibodies (17b, 48d and F425-A1g8). Recombinant CDV vectors encoding two chimeric Envs have been created. One is expressed most abundantly in transfected cells and contained the VSV SP and CDV TM-CT. The second is a highly fusogenic Env that contained the CDV SP and CDV CT. Both vectors expressed Env and are being further characterized in vitro and in vivo.

## Conclusion

Collectively, we have shown CDV can be used as a mucosal delivery vector for SIV-Env and that HIV-Env modifications can be made that improve cell surface expression of the trimeric glycoprotein containing structural determinants recognized by bnAbs.

